# IS*26* drives the dissemination of *bla*_CTX-M_ genes in an Ecuadorian community

**DOI:** 10.1128/spectrum.02504-23

**Published:** 2023-12-13

**Authors:** Liseth Salinas, Paúl Cárdenas, Jay P. Graham, Gabriel Trueba

**Affiliations:** 1 Universidad San Francisco de Quito, Colegio de Ciencias Biológicas y Ambientales, Instituto de Microbiología, Quito, Pichincha, Ecuador; 2 Environmental Health Sciences Division, University of California, Berkeley, California, USA; Peking University People's Hospital, Beijing, China

**Keywords:** *Escherichia coli*, *bla*_CTX-M_, IS*26*, horizontal gene transfer, antimicrobial resistance

## Abstract

The rapid dissemination of extended-spectrum β-lactamase (ESBL)-producing Enterobacterales, mainly *Escherichia coli* carrying *bla*_CTX-M_ genes, is a major public health concern due to its successful spread in hospital settings as well as among humans and animals in the community. We characterized ESBL-producing *E. coli* isolates from children and domestic animals in semirural communities of Ecuador to assess the contribution of horizontal gene transfer of the *bla*_CTX-M_ genes among *E. coli* isolates. From 20 selected *E. coli* isolates (from children and domestic animals) harboring *bla*_CTX-M_ allelic variants, we identified 16 plasmids carrying *bla*_CTX-M-55_ (*n* = 9), *bla*_CTX-M-65_ (*n* = 5), and *bla*_CTX-M-27_ (*n* = 2), as well as four chromosomes carrying *bla*_CTX-M-65_. The backbone structure of plasmids, including replication, maintenance, and plasmid transfer genes, and the synteny was conserved in all plasmids carrying the same *bla*_CTX-M_ allelic variant. In all plasmids and chromosomes, the *bla*_CTX-M_ genes were bracketed by two IS*26* transposable elements. This study highlights the critical role of the IS*26* transposable element for the current mobility of *bla*_CTX-M_ genes among plasmids or from plasmids to chromosomes, suggesting that IS*26-bla*_CTX-M_ brackets could be used to study *bla*_CTX-M_ transmission between humans, domestic animals, and the environment.

**IMPORTANCE:**

The horizontal gene transfer events are the major contributors to the current spread of CTX-M-encoding genes, the most common extended-spectrum β-lactamase (ESBL), and many clinically crucial antimicrobial resistance (AMR) genes. This study presents evidence of the critical role of IS*26* transposable element for the mobility of *bla*_CTX-M_ gene among *Escherichia coli* isolates from children and domestic animals in the community. We suggest that the nucleotide sequences of IS*26*-*bla*_CTX-M_ could be used to study *bla*_CTX-M_ transmission between humans, domestic animals, and the environment, because understanding of the dissemination patterns of AMR genes is critical to implement effective measures to slow down the dissemination of these clinically important genes.

## INTRODUCTION

The global spread of antimicrobial resistance (AMR) genes is a major threat that affects human health (1). Of particular concern is the dramatic dissemination of extended-spectrum β-lactamase (ESBL)-producing Enterobacterales, with *Escherichia coli* carrying CTX-M enzyme being the most common (2, 3). Strains carrying ESBLs are resistant to third- or later-generation cephalosporins (4), antimicrobials used in hospitals for patients suffering from life-threatening infections (5).

The origin of CTX-M-encoding genes has been traced to the chromosomes of several species of *Kluyvera* genus (6 – 8) from which these *bla*_CTX-M_ genes have disseminated to other Gammaproteobacteria; it was detected for the first time in *E. coli* in 1991 (9). Currently, more than 260 *bla*_CTX-M_ allelic variants have been identified and clustered into five groups (CTX-M-1, CTX-M-2, CTX-M-8, CTX-M-9, and CTX-M-25) based on their amino acid sequences (10, 11). In recent years, CTX-M has been the most common ESBL (2, 3) found in a large number of clinically significant bacteria (12 – 15), and bacteria from human communities (16, 17) and domestic animals (13, 18).

The rapid dissemination of *bla*_CTX-M_ genes deserves close attention. In a previous study, we found 16 *E. coli* clonal groups (72 *E. coli* isolates involved, of which 95% carried *bla*_CTX-M_ genes) associated with either humans or domestic animals in semirural communities in Ecuador, of which 21 *E. coli* strain pairs (14%) showed evidence of recent transmission between domestic animals and humans (18). In this study, we assessed the contribution of horizontal gene transfer of the *bla*_CTX-M_ genes among *E. coli* from humans and domestic animals in these communities.

## RESULTS

From the 20 selected *bla*_CTX-M_ allelic variant carrier *E. coli* isolates, we identified 16 plasmids carrying *bla*_CTX-M-55_ (CTX-M-1 group; *n* = 9), *bla*_CTX-M-65_ (CTX-M-9 group; *n* = 5), and *bla*_CTX-M-27_ (CTX-M-9 group; *n* = 2) and four chromosomes carrying *bla*_CTX-M-65_.

### Conjugation experiments

Conjugative assays revealed that all 16 *bla*_CTX-M_ allelic variants carried by plasmids were successfully transferable to the recipient *E. coli* TOP10 strain. All transconjugants showed the ESBL phenotype and were resistant to ampicillin (AM; 10 µg), cefazolin (CZ; 30 µg), and cefotaxime (CTX; 30 µg), but susceptible to amoxicillin-clavulanate (AMC; 20 per 10 μg), ciprofloxacin (CIP; 5 µg), imipenem (IPM; 10 µg), tetracycline (TE; 30 µg), and trimethoprim-sulfamethoxazole (SXT; 1.25 per 23.75 μg) (Table S1). Consistent with the sequencing results, four *bla*_CTX-M_ gene variant carrier *E. coli* isolates could not produce transconjugants because *bla*_CTX-M_ genes were identified on their chromosomes.

### Plasmid sequence analysis

**Fig 1:**
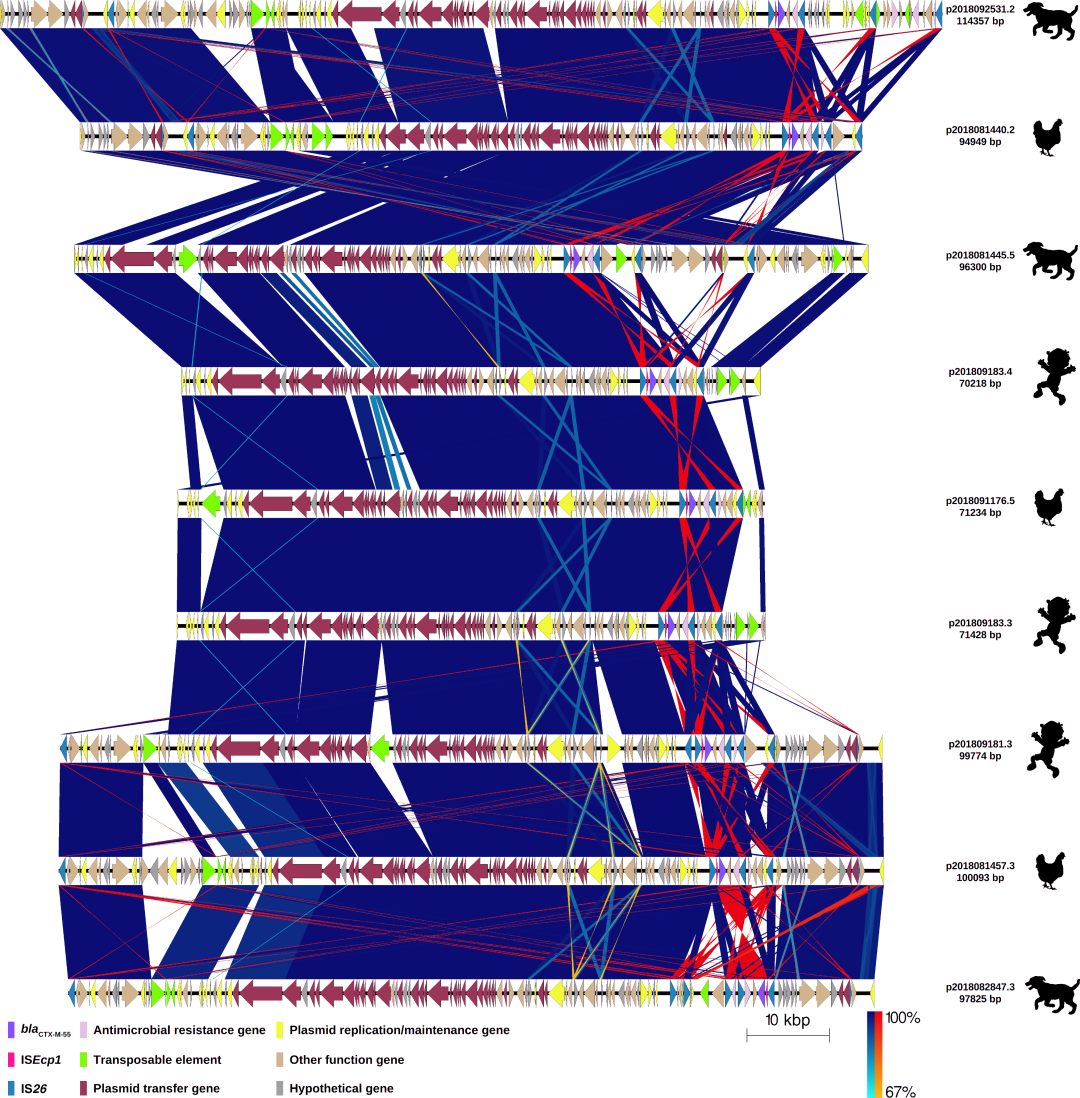
Comparison of nine plasmids carrying *bla*_CTX-M-55_ gene variant of extended-spectrum β-lactamase-producing *Escherichia coli* isolates from children, chickens, and dogs. Labels show the plasmid ID assigned based on the host ID followed by its isolate number and length of the plasmid carrying the *bla*_CTX-M-55_ allelic variant. The origin of the isolate harboring the plasmid is shown by a figure in black (child, chicken, and dog). Each plasmid is represented by linear visualization, and coding sequences (CDSs) are represented by arrows. The direction of the arrow indicates the transcription direction of each CDS. CDSs are colored based on their functions. Blue and red shading areas between plasmids indicate the similarity of regions in the same and inverted directions, respectively, according to BLASTn. The percentage of sequence similarity is shown according to a color gradient.

**Fig 2:**
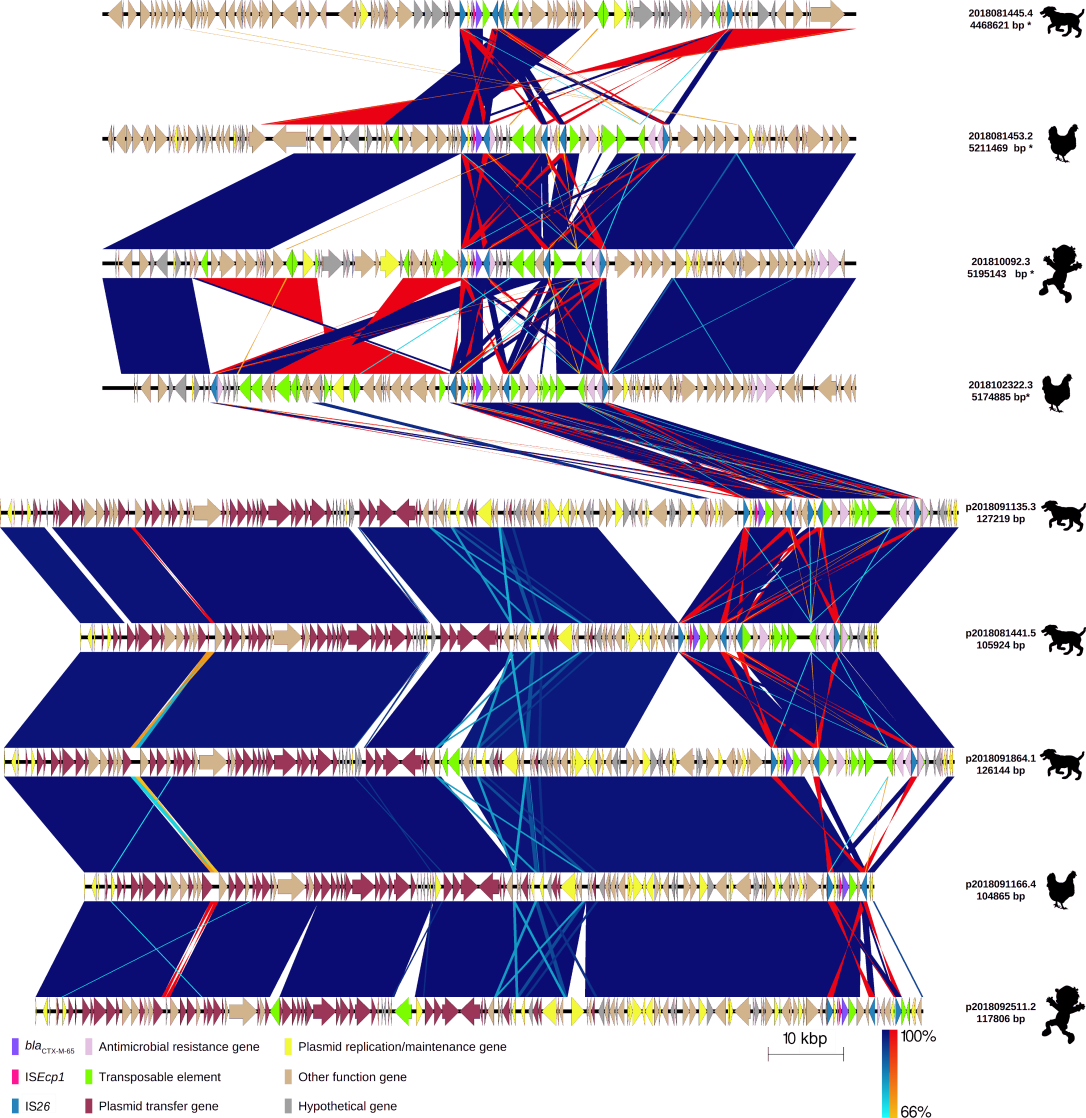
Comparison of four chromosome fragments (100,000 pb) and five plasmids carrying *bla*_CTX-M-65_ gene variant of extended-spectrum β-lactamase-producing *Escherichia coli* isolates from children, chickens, and dogs. Labels show the plasmid and chromosome ID assigned based on the host ID followed by its isolate number and length of chromosome* and plasmid carrying *bla*_CTX-M-65_ allelic variant. The origin of the isolate harboring the plasmid is shown by a figure in black (child, chicken, and dog). Each chromosome fragment or plasmid is represented by linear visualization, and coding sequences (CDSs) are represented by arrows. The direction of the arrow indicates the transcription direction of each CDS. CDSs are colored based on their functions. Blue and red shading areas between sequences indicate the similarity of regions in the same and inverted directions, respectively, according to BLASTn. The percentage of sequence similarity is indicated according to a color gradient.

**Fig 3:**
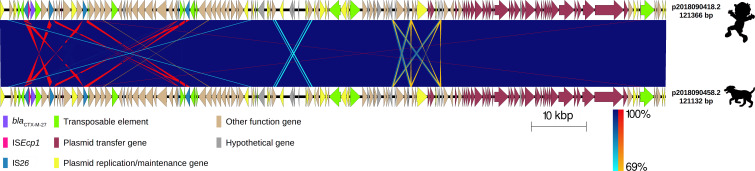
Comparison of two plasmids carrying *bla*_CTX-M-27_ gene variant of extended-spectrum β-lactamase-producing *Escherichia coli* isolates that were part of a clonal relationship with 0 SNPs in their core genomes. Labels show the plasmid ID assigned based on the host ID followed by its isolate number and length of the plasmid carrying the *bla*_CTX-M-27_ allelic variant. The origin of the isolate harboring the plasmid is shown by a figure in black (child and dog). Each plasmid is represented by linear visualization and coding sequences (CDSs) represented by arrows. The direction of the arrow indicates the transcription direction of each CDS. CDSs are colored based on their functions. Blue and red shading areas between plasmids indicate the similarity of regions in the same and inverted directions, respectively, according to BLASTn. The percentage of sequence similarity is indicated according to a color gradient.

The origins, sizes, and replicons of plasmids are summarized in Table 1. The backbone structure of plasmids (i.e., replication, maintenance, and plasmid transfer genes) and the synteny were conserved in all plasmids carrying the same *bla*_CTX-M_ allelic variant (Fig. 1 to 3). Plasmids carrying *bla*_CTX-M-65_, *bla*_CTX-M-55_, or *bla*_CTX-M-27_, however, formed distinct clusters (Fig. S1). A BLASTn analysis of one plasmid representative of each of our *bla*_CTX-M-55_ clusters (four in total) revealed high identity (99.28%–99.86%) and high query coverage (68%–100%) with three plasmids in two *E. coli* strains (MG197492.1, source: pig, isolation year: 2014; and MG197502.1, source: human, isolation year: 2013) and a *Klebsiella pneumoniae* strain (CP076034.1, source: human, isolation year: 2022), all from China (Fig. S2). The two plasmids carrying *bla*_CTX-M-65_ (one from each cluster) showed high identity (99.81%) and query coverage (90%–100%) with two plasmids in an *E. coli* strain (CP047572.1, source: human, isolation year: 2019) and a *Salmonella enterica* strain (CP074344.1, source: human, isolation year: 2010) isolated in Singapore and Perú, respectively (Fig. S3). In all cases, the plasmids from GenBank carried the same *bla*_CTX-M_ allelic variant as the plasmids from Ecuador.

**TABLE 1:** Length, plasmid types, and origin of plasmids and chromosomes carrying *bla*_CTX-M_ genes

Sequence ID	Origin of *E. coli* isolate	Allelic variant *bla*_CTX-M_	Size (bp)	Plasmid type
p201809183.4	Child	*bla*_CTX-M-55_	70,218	IncFII(pHN7A8)
p2018091176.5	Chicken	*bla*_CTX-M-55_	71,234	IncFII(pHN7A8) - IncFII(p96A)
p201809183.3	Child	*bla*_CTX-M-55_	71,428	IncFII(pHN7A8) - IncFII(p96A)
p2018081440.2	Chicken	*bla*_CTX-M-55_	94,949	IncFII(pHN7A8) - IncFII(p96A) - ColE10 - ^*a*^IncN
p2018081445.5	Dog	*bla*_CTX-M-55_	96,300	IncFII(pHN7A8) - ^*a*^IncN
p2018082847.3	Dog	*bla*_CTX-M-55_	97,825	IncFII(pHN7A8) - IncFII(p96A) - ColE10 - ^*a*^IncN
p201809181.3	Child	*bla*_CTX-M-55_	99,774	IncFII(pHN7A8) - IncFII(p96A) - ColE10 - ^*a*^IncN
p2018081457.3	Chicken	*bla*_CTX-M-55_	100,093	IncFII(p96A) - IncFIC(FII) - ColE10 - IncN
p2018092531.2	Dog	*bla*_CTX-M-55_	114,357	IncFII(pHN7A8) - IncFII(p96A) - IncX1 - IncX9 - ColE10 - IncN
p2018091166.4	Chicken	*bla*_CTX-M-65_	104,865	IncIγ - IncFII(pECLA)
p2018081441.5	Dog	*bla*_CTX-M-65_	105,924	IncI1 - IncFII(pECLA)
p2018092511.2	Child	*bla*_CTX-M-65_	117,806	IncIγ - IncFII(pECLA)
p2018091864.1	Dog	*bla*_CTX-M-65_	126,144	IncIγ - IncFII(pECLA)
p2018091135.3	Dog	*bla*_CTX-M-65_	127,219	IncIγ - IncFII(pECLA)
2018081445.4	Dog	*bla*_CTX-M-65_	4,468,621	-
2018102322.3	Chicken	*bla*_CTX-M-65_	5,174,885	-
201810092.3	Child	*bla*_CTX-M-65_	5,195,143	-
2018081453.2	Chicken	*bla*_CTX-M-65_	5,211,469	-
p2018090418.2	Child	*bla*_CTX-M-27_	121,366	IncFIB(AP001918) – IncFII
p2018090458.2	Dog	*bla*_CTX-M-27_	121,132	IncFIB(AP001918) – IncFII

^*a*^ Two copies of this plasmid type are carried by the plasmid.

### Genetic environment of *bla*_CTX-M_ genes

**Fig 4:**
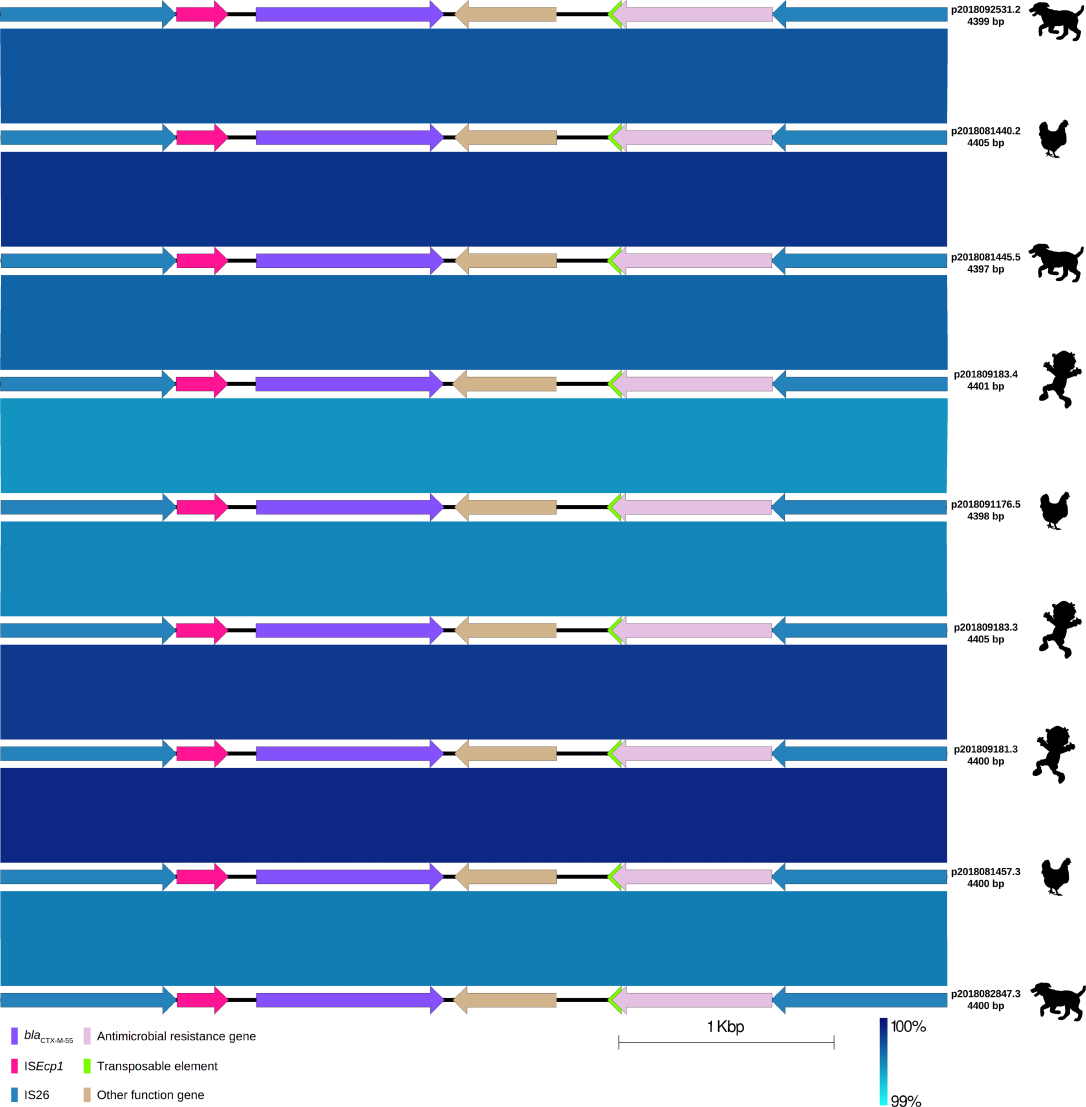
Comparison of nine IS*26-bla*_CTX-M-55_ brackets of extended-spectrum β-lactamase-producing *Escherichia coli* isolates from children, chickens, and dogs. Labels show plasmid ID (harboring the IS*26-bla*_CTX-M_ bracket) assigned based on the host ID followed by its isolate number and length of the IS*26-bla*_CTX-M-55_ bracket. The origin of the isolate harboring the plasmid is shown by a figure in black (child, chicken, and dog). Each IS*26-bla*_CTX-M-55_ bracket is represented by linear visualization, and coding sequences (CDSs) are represented by arrows. The direction of the arrow indicates the transcription direction of each CDS. CDSs are colored based on their functions. Blue shading areas between plasmids indicate the similarity of regions in the same direction according to BLASTn. The percentage of sequence similarity is indicated according to a color gradient.

**Fig 5:**
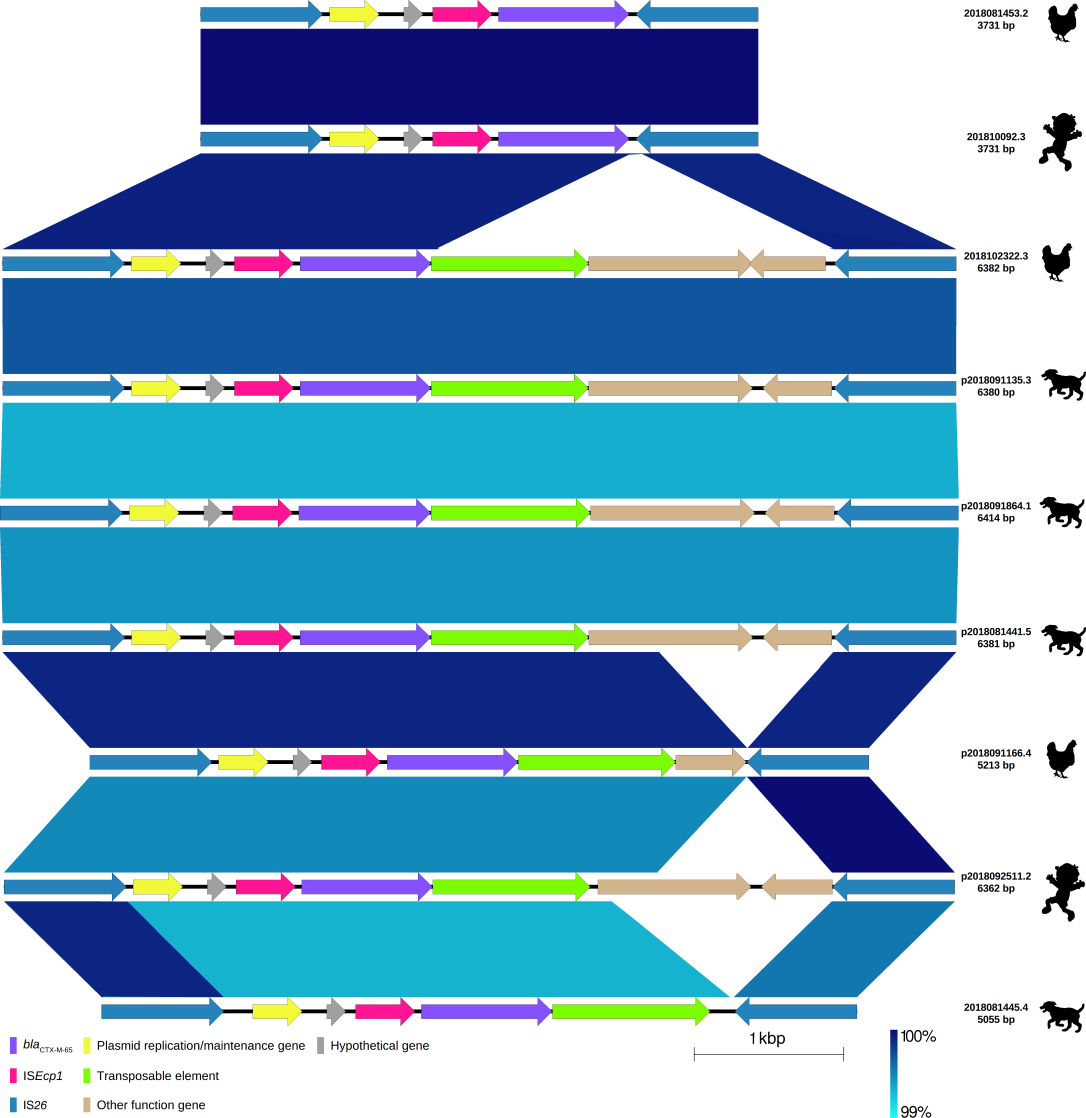
Comparison of nine IS*26-bla*_CTX-M-65_ brackets of extended-spectrum β-lactamase-producing *Escherichia coli* isolates from children, chickens, and dogs. Labels show the plasmid or chromosome ID (harboring the IS*26-bla*_CTX-M_ bracket) assigned based on the host ID followed by its isolate number and length of the IS*26-bla*_CTX-M-65_ bracket. The origin of the isolate harboring the plasmid or chromosome is shown by a figure in black (child, chicken, and dog). Each IS*26-bla*_CTX-M-65_ bracket is represented by linear visualization, and coding sequences (CDSs) are represented by arrows. The direction of the arrow indicates the transcription direction of each CDS. CDSs are colored based on their functions. Blue shading areas between plasmids indicate the similarity of regions in the same direction according to BLASTn. The percentage of sequence similarity is indicated according to a color gradient.

In all the plasmids, the *bla*_CTX-M-55_ gene was bracketed by two IS*26* transposable elements and located 127 bp downstream of a fragment of IS*ECp1* insertion sequence (243 bp; 14.7% coverage) truncated by IS*26,* and 46 bp upstream of the *wbuC* gene, which codes a cupin fold metalloprotein. Downstream of the *wbuC* gene, the Tn*A* and *bla*_TEM_ genes were found, and both were truncated by IS*26* (Fig. 4). This structure was the same (99% identity) for all nine *bla*_CTX-M-55_-carrying plasmids (Fig. 4). We use the term IS*26-bla*_CTX-M_ bracket to indicate the nucleotide sequence containing the *bla*_CTX-M_ gene that is flanked by two IS*26*s. In the plasmid from one isolate (ID: 2018082847.3), the IS*26-bla*_CTX-M_ bracket, containing *bla*_CTX-M-55_ and identical genes, was in the opposite direction, indicating inversion caused most probably by recombination or transposition of both IS*26*s (Fig. 1). The nine *bla*_CTX-M-65_ gene variants were detected in five plasmids and four chromosomes. Similar to the case of *bla*_CTX-M-55_, the *bla*_CTX-M-65_ gene in all cases was bracketed by two IS*26*s (Fig. 5): in three plasmids (from 2018091135.3, 2018091864.1, and 2018081441.5 isolates) and one chromosome (from 2018102322.3 isolate), IS*26-bla*_CTX-M_ brackets contained the same genes: *fipA* gene encoding a conjugal transfer inhibition protein, a hypothetical gene, IS*Ecp1* fragment, *bla*_CTX-M-65_ gene, IS*102* insertion sequence, a gene encoding a TonB-dependent receptor, and a gene encoding PAS domain-containing protein; in the two chromosomes (from 2018081453.2 and 201810092.3 isolates), the IS*26-bla*_CTX-M_ bracket contained fewer of the same genes in the same order: *fipA* gene encoding a conjugal transfer inhibition protein, the hypothetical gene, IS*Ecp1* fragment, and *bla*_CTX-M-65_ gene. Although the IS*26-bla*_CTX-M_ bracket contained the same genes in the same order, some of the genes were located at different distances from each other: IS*26 – fipA* gene (44 bp: 2018081453.2, 201810092.3, p2018102322.3, p2018091864.1, and p2018081441.5; 43 bp: p2018091135.3), *fipA* gene – hypothetical gene (64 bp: 2018081453.2, 201810092.3, p2018091135.3, and p2018091864.1; 63 bp: p2018102322.3 and p2018081441.5), gene encoding a TonB-dependent receptor – gene encoding PAS domain-containing protein (67 bp: p2018091135.3 and p2018081441.5; 68 bp: p2018091864.1; 24 bp: p2018102322.3), gene encoding PAS domain-containing protein – IS*26* (14 bp: p2018091135.3, p2018091864.1 and p2018081441.5; 57 bp: p2018102322.3).

### IS*26-bla*_CTX-M_ bracket similarity search

The IS*26-bla*_CTX-M-55_ bracket from p2018081440.2 showed similarity with sequences from six different species in the GenBank: *E. coli* (*n* = 69), *K. pneumoniae* (*n* = 12), *S. enterica* (*n* = 10), *Salmonella* sp. (*n* = 5), *Escherichia albertii* (*n* = 2), *Acinetobacter baumannii* (*n* = 1), and *Citrobacter freundii* (*n* = 1), with a query coverage of 100% and identities ranging from 99.84% to 99.91%. The IS*26-bla*_CTX-M-65_ bracket from p2018091135.3 showed similarity with sequences from four different species: *S. enterica* (*n* = 46), *Proteus mirabilis* (*n* = 26), *E. coli* (*n* = 24), and *K. pneumoniae* (*n* = 3), with a query coverage between 95% and 100% and identities between 96.33% and 99.67%. Whereas the IS*26-bla*_CTX-M-65_ bracket from p201810092.3 showed similarities with sequences from eight different species: *E. coli* (*n* = 46), *P. mirabilis* (*n* = 24), *S. enterica* (*n* = 21), *K. pneumoniae* (*n* = 3), *Kluyvera intermedia* (*n* = 2), *Enterobacter hormaechei* (*n* = 1), *Escherichia fergusonii* (*n* = 1), and *Klebsiella aerogenes* (*n* = 1), with a query coverage between 56% and 100% and identities between 99.76% and 99.95%.

To assess the single-nucleotide polymorphisms (SNPs) in the IS*26-bla*_CTX-M_ brackets, we carried out pairwise SNP analysis between the three brackets with the sequences showing the highest similarity in BLASTn analyses. For IS*26-bla*_CTX-M-55_ bracket from p2018081440.2, we identified 88 (88%) that presented 2 SNPs and 12 (12%) showing 3 SNPs. For the IS*26-bla*_CTX-M-65_ bracket from p2018091135.3, the pairwise SNPs analysis showed 34 (34.34%), 56 (56.57%), 7 (7.07%), and 2 (2.02%) sequences with 1, 2, 3, and 4 SNPs, respectively. Interestingly, 1 (1.01%) and 98 (98.99%) of the 99 match sequences in the IS*26-bla*_CTX-M-65_ bracket from p201810092.3 showed 1 and 0 SNPs, respectively.

### Phylogenetic analysis of plasmids and IS*26-bla*_CTX-M_ brackets

**Fig 6:**
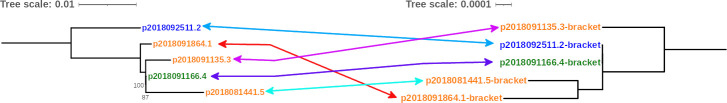
Comparative phylogenetic analysis of complete sequences of plasmids carrying *bla*_CTX-M-65_ allelic variant with their harbored IS*26-bla*_CTX-M_ bracket. The evolutionary history was inferred using maximum-likelihood phylogenetics with a general time reversible tree built using the genetic distance. The phylogenetic tree on the left was based on complete sequences of plasmids, whereas the tree on the right was based on IS*26-bla*_CTX-M_ sequences. Labels show the isolate ID assigned based on the host ID followed by its isolate number. The origin of the isolate harboring the plasmid is indicated by font colors (child: blue; dog: orange; chicken: green). Colored arrows relate the plasmid to its corresponding IS*26-bla*_CTX-M_ bracket. Bootstrap values (>80) based on 100 replications are shown at the tree nodes.

**Fig 7:**
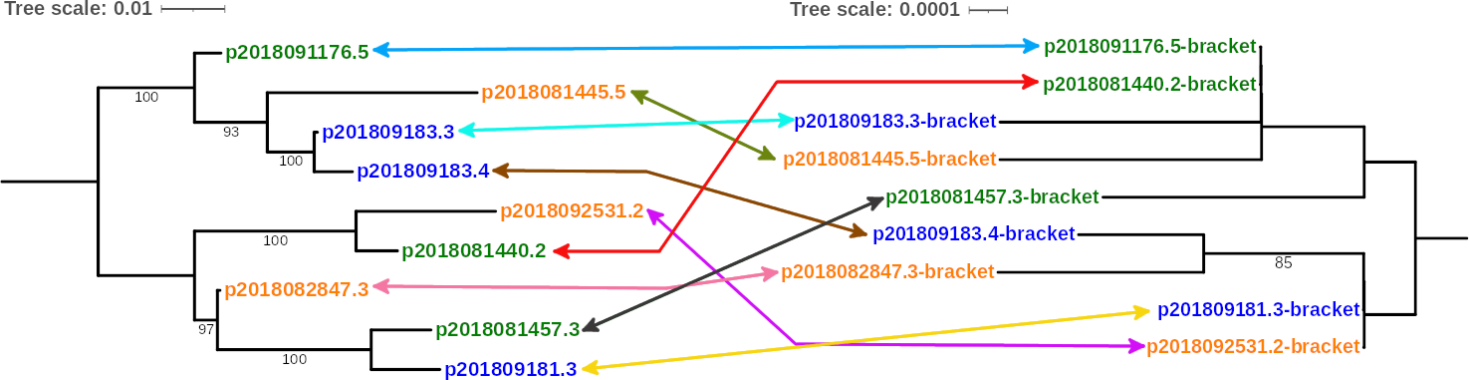
Comparative phylogenetic analysis of complete sequences of plasmids carrying *bla*_CTX-M-55_ allelic variant with their harbored IS*26-bla*_CTX-M_ bracket. The evolutionary history was inferred using maximum-likelihood phylogenetics with a general time reversible tree built using the genetic distance. The phylogenetic tree on the left was based on complete sequences of plasmids, whereas the tree on the right was based on IS*26-bla*_CTX-M_ sequences. Labels show the isolate ID assigned based on the host ID followed by its isolate number. The origin of the isolate harboring the plasmid is indicated by font colors (child: blue; dog: orange; chicken: green). Colored arrows relate the plasmid to its corresponding IS*26-bla*_CTX-M_ bracket. Bootstrap values (>80) based on 100 replications are shown at the tree nodes.

To explore the possibility of IS*26-bla*_CTX-M_ bracket mobilization among different plasmids, we compared the topology of the maximum likelihood phylogenetic trees of the plasmids and the IS*26-bla*_CTX-M_ brackets carrying either *bla*_CTX-M-65_ or *bla*_CTX-M-55_. Even though some plasmid clustering was concordant with IS*26-bla*_CTX-M_ brackets (e.g., plasmids p2018091166.4 and p2018081441.5 show a common ancestor and p2018082847.3 and p201809181.3 also share a common ancestor), there were many cases where the clustering of plasmids and IS*26-bla*_CTX-M_ brackets were discordant (e.g., plasmids p2018092531.2 and p2018081440.2 share a recent common ancestor while their IS*26-bla*_CTX-M_ brackets share a recent ancestor with IS*26-bla*_CTX-M_ brackets from other plasmids, p2018092531.2 with p201809181.3, and p2018081440.2 with 2018091176.5) (Fig. 6 and 7). These results suggest that many plasmids have not co-evolved for some time with their respective IS*26-bla*_CTX-M_ brackets.

### Plasmid evolutionary rate

To determine the rate of plasmid (carrying *bla*_CTX-M_ genes) evolution, we took advantage of four clonal *E. coli* strains (2018090418.2 and 2018090458.2: 0 SNPs; 2018091135.3 and 2018081441.5: 90 SNPs) isolated during the same period in the same community (18). This plasmid comparison showed a highly conserved structure with an extremely high nucleotide identity (28 SNPs). There were no plasmid rearrangements, gene insertions, or deletions for plasmids from *E. coli* strains with 0 SNPs in their core genomes. The regions with lower similarity (69%) corresponded to duplicated sequences of hypothetical genes and intergenic spaces. The plasmid sizes were 121,366 bp and 121,132 bp for plasmids p2018090418.2 and p2018090458.2, respectively (Fig. 3; Table S2). IS*26* also bracketed the two *bla*_CTX-M-27_ genes*,* and this region presented 100% identity. The two plasmids carrying *bla*_CTX-M-65_ from *E. coli* strains with 90 SNPs in their core genomes also showed a conserved structure with high nucleotide identity (209 SNPs). There were no rearrangements found; however, gene insertion and deletion regions were identified. The lower similarity (71%) corresponded to duplicated sequences of IS*26* and intergenic spaces. The plasmid sizes were 127,219 bp and 105924 bp from 2018091135.3 and 2018081441.5 isolates, respectively (Fig. 2; Table S2).

To further strengthen the results of this analysis, we determined the number of SNPs and length differences between plasmids sequenced in duplicate, as well as the thresholds to define variations due to inherent variations of sequencing and bioinformatics analyses. The mean SNP difference was 0.06%, and the mean length difference was 0.10% (Table S2).

## MATERIALS AND METHODS

### ESBL-producing *E. coli* whole genomes

In a previous study that aimed to study the transmission of cephalosporin-resistant *E. coli* between domestic animals and humans, we analyzed *E. coli* strains that showed high chromosomal similarity (18). To study horizontal gene transfer, we selected 125 ESBL-producing *E. coli* (from domestic animals and humans) carrying the most common *bla*_CTX-M_ allelic variants identified in these communities (18). We used the ResFinder database (19), with 90% minimum match and 60% minimum length (18). Sixty-nine carried the *bla*_CTX-M-55_ allelic variant (children = 22, dogs = 20, chickens = 27) and 56 carried the *bla*_CTX-M-65_ allelic variant (children = 7, dogs = 34, chickens = 15).

### Characterization of *bla*_CTX-M_ carrier contigs

Mobile genetic elements of 125 *bla*_CTX-M_ gene variant carrier contigs were identified using the command-line version of MobileElementFinder 1.0.3 (20) with 80% minimum match and 10% minimum length. Then, *bla*_CTX-M-55_ and *bla*_CTX-M-65_ allelic variant carrier contigs were separately aligned in Unipro UGENE (21) to establish them into groups based on the similarity of their nucleotide sequences. The *bla*_CTX-M-55_ gene carrier contigs were classified into six groups, whereas the *bla*_CTX-M-65_ gene carrier contigs were classified into eight groups. From the established groups in which there were contigs from ESBL-producing *E. coli* whole genomes isolated from more than one species, we randomly selected one isolate from each species for further analyses. Additionally, to determine the rate of plasmid (carrying *bla*_CTX-M_ genes) evolution, we chose four contigs of four different ESBL-producing *E. coli* whole genomes: two with 0 SNPs in their core genomes (carrying *bla*_CTX-M-27_ allelic variant) and two with 90 SNPs in their core genomes (carrying *bla*_CTX-M-65_ allelic variant) (18).

### DNA extraction of *bla*_CTX-M_ gene carrier plasmids

Each of the 20 selected *bla*_CTX-M_ allelic variant carrier *E. coli* isolates was reactivated on MacConkey Lactose agar (Difco) supplemented with ceftriaxone (2 mg/L) overnight at 37°C, after which one colony was selected and inoculated into 2 mL of Lysogeny Broth (LB) supplemented with ceftriaxone (2 mg/L) with shaking at 250 rpm at 37°C for 9 h. Then, 3 mL of fresh LB media with antibiotic were added to the culture at 37°C for 12–16 h while shaking at 250 rpm. Plasmid extraction from the 20 isolates was performed in duplicate using Pure Yield Plasmid Miniprep System (Promega) according to the protocol provided by the manufacturer. Duplicates were placed into a single microtube before being freeze-dried and resuspended in nuclease-free water to achieve a minimum plasmid DNA concentration of 53 ng/µL. Extracted plasmid DNA concentrations were measured using a Qubit 1× dsDNA High Sensitivity assay kit and a Qubit 4.0 fluorometer (Thermo Fisher Scientific).

### Genomic DNA extraction

For the four isolates whose sequences could not be circularized or in which the *bla*_CTX-M_ allelic variant could not be identified after sequencing and assembly, genomic DNA extraction was performed using 12–16 h cultures obtained as mentioned above, using DNeasy Blood & Tissue kit (Qiagen). DNA was eluted in nuclease-free water, and a minimum DNA concentration of 53 ng/µL was obtained, measured using a Qubit 1X dsDNA High Sensitivity assay kit and a Qubit 4.0 fluorometer (Thermo Fisher Scientific).

### Conjugation experiments

Conjugation assays were performed to evaluate the conjugative capacity of *bla*_CTX-M_ carrier plasmids. The 20 selected *bla*_CTX-M_ allelic variant carrier *E. coli* isolates were used as donors and *E. coli* TOP10 (Invitrogen) resistant to rifampin as the recipient (22). Prior to conjugation experiments, the phenotypic AMR profile of each donor strain was confirmed against the same 12 antimicrobials used in our previous study (18) using the disk diffusion method according to the Clinical and Laboratory Standards Institute guidelines (23). Among the 12 antimicrobials, we used ceftazidime (CAZ; 30 µg), CTX (30 µg), cefepime (FEP; 30 µg), and AMC (20 per 10 µg), with which we carried out the double-disk synergy test (24). Phenotypic expression of ESBL was evaluated by placing a disk of AMC surrounded by disks of CAZ, CTX, and FEP (30 mm apart, center to center). An extension of the edge of the CAZ, CTX, or FEP inhibition zone toward AMC disk as a keyhole effect was interpreted as positive for the ESBL phenotype (24, 25). For each conjugation experiment, the donor and recipient strains were grown in LB at 37°C for 18 h, and the strains in the logarithmic growth phase were mixed and incubated at 37°C for 18 h. Transconjugants were selected by the spread plate method onto LB agar-containing ceftriaxone (2 mg/L) and rifampin (100 µg/mL) as previously described (22). The phenotypic expression of ESBL by DDST and antimicrobial phenotypic profile by disk diffusion of transconjugants were evaluated to determine the acquired AMR.

### MinION library preparation and sequencing

According to the manufacturer’s instructions, library preparation was performed using the Rapid Barcoding Sequencing Kit (SQK-RBK004) (Oxford Nanopore Technologies). The constructed libraries were loaded into R9.4.1 (FLO-MIN106D) flow cells and sequenced on a MinION Mk1B sequencing device for approximately 24 h using the MinKNOW software 22.03.5 (Oxford Nanopore Technologies). We sequenced a random selection of three plasmid DNA samples twice, obtained from the same bacterial cultures, to determine the intrinsic variations of sequencing and bioinformatic analyses. Basecalling was carried out with Guppy 6.0.6 (https://community.nanoporetech.com) in a fast basecalling model. Raw data were demultiplexed, and adapters and barcodes were trimmed using Porechop 0.2.4 with default parameters (26). Then, raw reads were filtered with Filtlong 0.2.1 using a minimum read length threshold of 1 kbp and keeping 95% of the best reads (measured by bases). Filtered reads metrics were assessed using NanoPlot 1.40.0 (27).

### Assembly of plasmids and chromosomes carrying *bla*_CTX-M_ gene

*De novo* assembly of complete plasmids and chromosomes with filtered reads was carried out using Flye assembler 2.8.1-b1676 (28). Different assembly parameters were evaluated to optimize the assembly of the circular sequences of interest, due to the unknown plasmid size and possible contamination with chromosomal DNA in the plasmid DNA samples. The genome-size option was set at 0.1, 1, 2, 3, 4, and 5 m each, with the asm-coverage option set at 10, 15, 20, 30, 40, and 50, with all combinations. The plasmids option was specified to allow recovery of unassembled short plasmids. Additionally, the meta-assembly option (29) was also assessed.

### *bla*_CTX-M_ gene variant carrier plasmid and chromosome annotation

AMR genes and plasmid types were identified with the Resfinder (19) and PlasmidFinder (30) databases, respectively, using ABRicate tool 1.0.1 (31) in all plasmids and chromosomes circularized. Each *bla*_CTX-M_ gene variant carrier plasmid and chromosome was rotated with the task fixstart of Circlator tool 1.5.5 (32) and a fasta file with 7171 ancestral sequences of the most common replication initiators (33) to fix the start position of each plasmid and chromosome. As plasmids usually have more than one replication initiator, they were aligned with MAFFT algorithm and manually modified to establish the same replication origin in cases where possible in Unipro UGENE 40.1 (21). For the plasmid sequences of the DNA samples sequenced twice, we used Unipro UGENE to align them and obtain the consensus sequences using the Levitsky algorithm. The number of SNPs between plasmids sequenced twice and between plasmids from clonal *E. coli* strains was determined using Snippy 4.6.0 (34). Each *bla*_CTX-M_ gene variant carrier plasmid and chromosome was annotated with the National Center for Biotechnology Information (NCBI) Prokaryotic Genome Annotation Pipeline (PGAP) (35). The output GenBank file was manually curated using data obtained from different annotation tools. Mobile genetic elements were identified using the command line version of MobileElementFinder 1.0.3 (20), and AMR genes and plasmid types were again predicted after rotation of sequences with the Resfinder and PlasmidFinder databases, respectively. The genomic structure comparison among plasmids and chromosomes and among IS*26-bla*_CTX-M_ brackets was performed according to BLASTn using Easyfig 2.2.2 (36).

### Similarity of IS*26-bla*_CTX-M_ brackets

The IS*26-bla*_CTX-M_ bracket sequences similarity search was carried out with the IS*26-bla*_CTX-M-55_ bracket (from p2018081440.2), and with the two most common IS*26-bla*_CTX-M-65_ brackets identified (from p2018091135.3 and p201810092.3, respectively), using BLASTn without inclusion or exclusion parameters. All 100 match sequences for each of the three IS*26-bla*_CTX-M_ brackets, excluding synthetic constructs, were selected. We used Unipro UGENE 40.1 to convert sequences to their reverse complement as necessary to ensure that all selected sequences are in the same orientation with respect to the IS*26-bla*_CTX-M_ brackets. Then, the number of SNPs between each of the selected sequences and their respective IS*26-bla*_CTX-M_ brackets was determined using Snippy 4.6.0.

### Phylogenetic analyses

To investigate the possibility of IS*26-bla*_CTX-M_ bracket mobilization among different plasmids, we constructed maximum likelihood phylogenetic trees of the plasmids and the IS*26-bla*_CTX-M_ brackets carrying *bla*_CTX-M-65_ or *bla*_CTX-M-55_. Due to inverted sequences in plasmids that concealed their phylogenetic relationships, we identified inverted DNA sequences using Easyfig 2.2.2 (36), and we manually placed these sequences in the same direction using Unipro UGENE 40.1 (21) before phylogenetic tree construction. We also carried out BLASTn analyses of one representative plasmid of each phylogenetic tree cluster obtained to identify the best match plasmids. Additionally, BLASTn analyses of IS*26-bla*_CTX-M-55_ bracket, and the most common IS*26-bla*_CTX-M-65_ bracket identified, were performed to establish the best match plasmid harbored by *Kluyvera* spp. Then, from the *Kluyvera* plasmid sequence more similar to the IS*26-bla*_CTX-M-65_ bracket, we carried out a new BLASTn comparison to select the four best match plasmid sequences to include them in a phylogenetic tree based on all of our plasmids carrying either *bla*_CTX-M-65_, *bla*_CTX-M-55_, or *bla*_CTX-M-27_. All maximum likelihood phylogenetic trees were performed with the general time reversible model using RaxML-NG 0.6.0 (37). The visualization and edition of phylogenetic trees were carried out using iTOL v6 (38) and GIMP 2.10 (https://www.gimp.org), respectively.

## DISCUSSION

Our results suggest that IS*26* mobilizes *bla*_CTX-M-65_, *bla*_CTX-M-55_, and *bla*_CTX-M-27_ allelic variants among different plasmids (Fig. 1 to 3). Even though some plasmids carrying the same *bla*_CTX-M_ gene share a more recent ancestor, which may suggest plasmid co-evolution with *bla*_CTX-M_ genes (Fig. S1), the phylogeny of the IS*26-bla*_CTX-M_ did not correspond to plasmid phylogeny (Fig. 6 and 7). Additional evidence of IS*26* contribution in *bla*_CTX-M_ mobility is the presence of the identical IS*26-bla*_CTX-M-65_ bracket in the plasmids of three different isolates and the chromosome of another isolate (Fig. 5). We also found evidence of different evolutionary trajectories of *bla*_CTX-M_ genes and plasmids; genes *bla*_CTX-M-27_ and *bla*_CTX-M-65_ belong to phylogenetic group 1, whereas gene *bla*_CTX-M-55_ belongs to phylogenetic group 9 (8). However, our results show that plasmids carrying *bla*_CTX-M-27_ share a more recent common ancestor with plasmids carrying *bla*_CTX-M-55_ than with plasmids carrying *bla*_CTX-M-65_ (Fig. S1). These observations suggest that the plasmids have exchanged IS*26-bla*_CTX-M_ brackets (through transposition or recombination) throughout their evolution. The large divergence in plasmids carrying *bla*_CTX-M_ genes is consistent with the notion that *bla*_CTX-M_ genes were associated with different plasmids that existed before the use of third-generation cephalosporins (14) (Table 1).

Our findings are also consistent with recent reports showing that IS*26* is extremely active, as transposable elements, mobilizing many important AMR genes (39). In our study, however, the IS*26-bla*_CTX-M-55_ brackets carried a fragment of the *bla*_TEM_ gene (in addition to the *bla*_CTX-M_ gene); seven of the nine plasmids carrying *bla*_CTX-M-55_ allelic variant showed the *fosA3* gene (coding for fosfomycin resistance) in the vicinity, whereas other plasmids showed a fragment of the *mef(B)* gene (coding for a macrolide efflux pump) in the vicinity (Fig. 1). Similarly, IS*26-bla*_CTX-M-65_ brackets showed more AMR genes: *fosA3*, *floR* (coding for chloramphenicol), *aph (4)-Ia* (coding for an aminoglycoside phosphotransferase), *aac (3)-Iva* (coding for gentamicin), and *ant(3″)-Ia* (coding for streptomycin) in the vicinity (Fig. 2).

Even though we were not able to observe direct transmission of a plasmid between *E. coli* from domestic animals and humans, all the plasmids carrying *bla*_CTX-M_ genes were conjugable, and domestic animals and humans shared many (65%, 13 of 20) of the IS*26-bla*_CTX-M_ brackets. These findings suggest that horizontal gene transfer events of diverse plasmids and *bla*_CTX-M_ genes outnumber clonal transmission events (among domestic animals and humans), as we found 14% of *bla*_CTX-M_ *E. coli* strains presented evidence of recent transmission between humans and domestic animals (18). These results suggest that the IS*26-bla*_CTX-M_ bracket involves a complex multi-step process of horizontal gene transfer in which transposons mobilize the *bla*_CTX-M_ among plasmids or from plasmids to chromosomes. These results agree with previous observations that some *bla*_CTX-M_ gene variants (and their contiguous regions) were associated with specific environments in Ecuador (40). In these cases, the only evidence of AMR-gene transmission is the presence of the highly similar IS*26-bla*_CTX-M_ brackets in different isolates in an epidemiological context compatible with this transmission. We acknowledge that because *E. coli* isolates were obtained from the same geographic region in Ecuador (18), the results may not be generalizable to other countries, although our IS*26-bla*_CTX-M_ brackets were highly similar to the sequences found in plasmids and chromosomes from GenBank, suggesting that IS*26-bla*_CTX-M_ brackets may play an important role in the dissemination of the *bla*_CTX-M_ gene through several bacterial species in different geographic regions.

In conclusion, the prevalence of CTX-M enzymes has increased dramatically since the mid- to late 2000s (3). ESBL-encoding genes were identified in plasmids present in *E. coli* strains isolated before the use of third-generation cephalosporins in 1981, suggesting that the *E. coli* acquisition of these genes had occurred in multiple independent events (14). The IS*26* transposable element is critical for the current mobility of these and other clinically crucial AMR genes. Our study suggests that the nucleotide sequences of IS*26-bla*_CTX-M_ brackets could be an important genetic structure to study *bla*_CTX-M_ transmission between humans, domestic animals, and the environment. We provide evidence for the complexity of the *bla*_CTX-M_ horizontal gene transfer and how this understanding can be applied to determine the dissemination of these genes in any community, animals, or environment. The amplification and sequencing of the DNA inside the brackets may be used to monitor the *bla*_CTX-M_ dynamics (increasing rates, allelic variant replacement, dissemination, etc.). Understanding the dissemination patterns of AMR genes is critical to implementing effective measures to slow down the dissemination of these clinically important genes.

## Data Availability

Assembled plasmids and chromosomes were deposited into the NCBI database under accession number PRJNA973083.

## SUPPLEMENTAL MATERIAL

Fig. S1 spectrum.02504-23-s0001.png Supplemental figure.

Fig. S2 spectrum.02504-23-s0002.png Supplemental figure.

Fig. S3 spectrum.02504-23-s0003.png Supplemental figure.

Tables S1 to S2 and legends of Fig. S1 to S3 spectrum.02504-23-s0004.docx Supplemental tables and legends of supplemental figures.

ASM does not own the copyrights to Supplemental Material that may be linked to, or accessed through, an article. The authors have granted ASM a non-exclusive, world-wide license to publish the Supplemental Material files. Please contact the corresponding author directly for reuse.
