# Supplementary figures and images for: IS26 drives the dissemination of bla CTX-M genes in an Ecuadorian community
Source: Microbiol Spectr. 2023 Dec 13;12(1):e02504-23. doi: 10.1128/spectrum.02504-23 (PMC10783052; doi:10.1128/spectrum.02504-23)

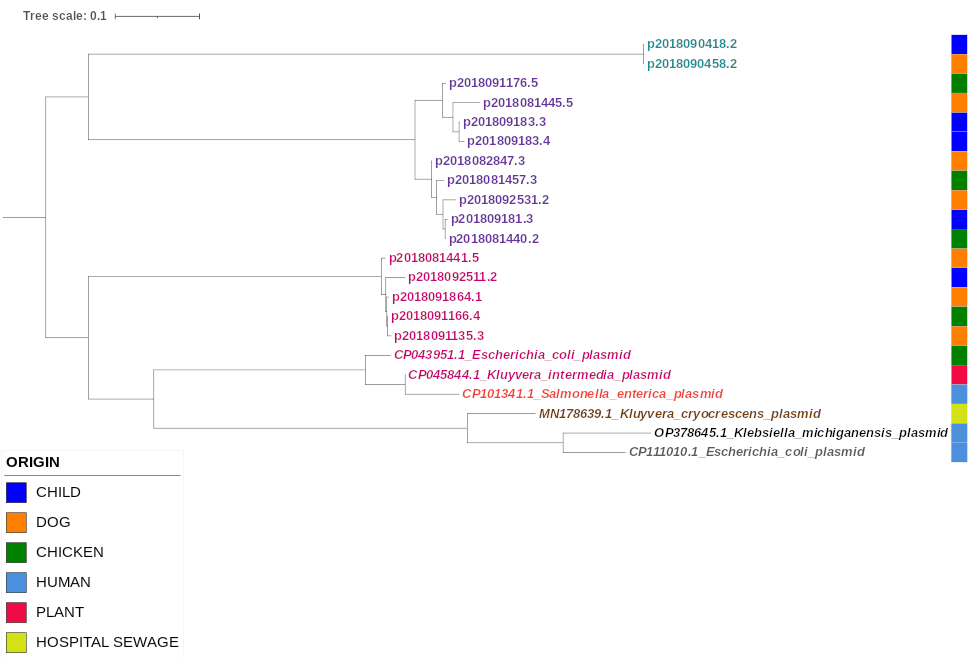

Supplement: Fig. S1 — Supplemental figure. [file spectrum.02504-23-s0001.png]

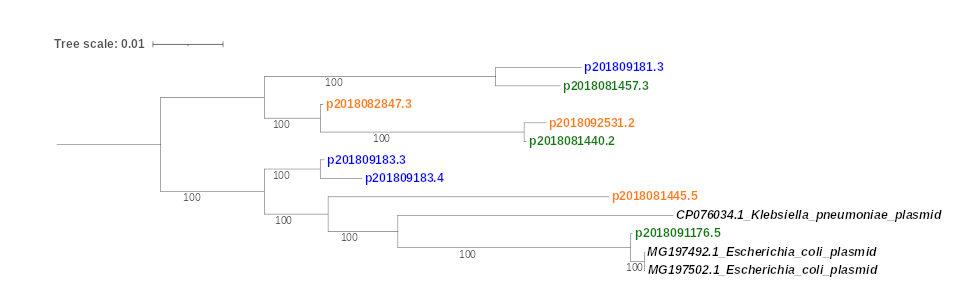

Supplement: Fig. S2 — Supplemental figure. [file spectrum.02504-23-s0002.png]

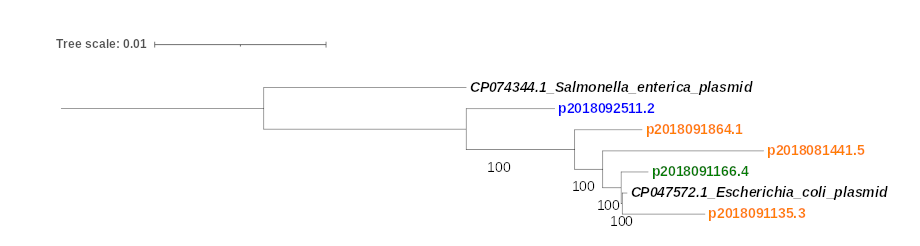

Supplement: Fig. S3 — Supplemental figure. [file spectrum.02504-23-s0003.png]
