# Supplementary material for: IS26 drives the dissemination of bla CTX-M genes in an Ecuadorian community
Source: Microbiol Spectr. 2023 Dec 13;12(1):e02504-23. doi: 10.1128/spectrum.02504-23 (PMC10783052; doi:10.1128/spectrum.02504-23)
Supplement: Tables S1 to S2 and legends of Fig. S1 to S3 — Supplemental tables and legends of supplemental figures. [file spectrum.02504-23-s0004.docx]

**Fig. S1.** Maximum-likelihood phylogenetic tree of complete sequences of plasmids carrying *bla*_CTX-M-55,_ *bla*_CTX-M-65,_ and *bla*_CTX-M-27_ allelic variants using closely related plasmid sequences based on BLASTn analyses. The evolutionary history was inferred using GTR tree built using the genetic distance. Labels show plasmid ID assigned based on the host ID followed by its isolate number. The origin of isolate harboring the plasmid is shown by a color strip. Allelic variant held by each plasmid is shown by font colors (*bla*_CTX-M-27_: turquoise; *bla*_CTX-M-55_: purple; *bla*_CTX-M-65_: fuchsia; *bla*_OXA-1_: red; *bla*_CTX-M-3_: brown; *bla*_KPC-3_: black; *bla*_KPC-2_: gray). CP043951.1, isolation year: 2017, location: China. CP045844.1, isolation year: 2017, location: China. CP101341.1, isolation year: 2013, location: China. MN178639.1, isolation year: 2019, location: China. OP378645.1, isolation year: 2018, location: EEUU. CP111010.1, isolation year: 2022, location: China.

**Fig. S2.** Maximum-likelihood phylogenetic tree of complete sequences of plasmids carrying *bla*_CTX-M-55_ allelic variant using closely related plasmid sequences based on BLASTn analyses. The evolutionary history was inferred using GTR tree built using the genetic distance. Labels show plasmid ID assigned based on the host ID followed by its isolate number. The origin of isolate harboring the plasmid is shown by font colors (child: blue; dog: orange; chicken: green; black: NCBI database). CP076034.1, isolation year: 2022, location: China, allelic variant: *bla*_CTX-M-55_. MG197492.1, isolation year: 2014, location: China, allelic variant: *bla*_CTX-M-55_. MG197502.1, isolation year: 2013, location: China, allelic variant: *bla*_CTX-M-55_. Bootstrap values (> 80) based on 100 replications are shown at the nodes of the tree.

**Fig. S3.** Maximum-likelihood phylogenetic tree of complete sequences of plasmids carrying *bla*_CTX-M-65_ allelic variant using closely related plasmid sequences based on BLASTn analyses. The evolutionary history was inferred using GTR tree built using the genetic distance. Labels show plasmid ID assigned based on the host ID followed by its isolate number. The origin of isolate harboring the plasmid is shown by font colors (child: blue; dog: orange; chicken: green; black: NCBI database). CP074344.1, isolation year: 2010, location: Perú, allelic variant: *bla*_CTX-M-65_. CP047572.1, isolation year: 2010, location: Singapore, allelic variant: *bla*_CTX-M-65_. Bootstrap values (> 80) based on 100 replications are shown at the tree nodes.

**Table S1.** Antimicrobial phenotypic profile of original isolates and their transconjugants.

| **Isolate ID** | **CZ** | | **CIP** | | **AM** | | **C** | | **IPM** | | **SXT** | | **GM** | | **CAZ** | | **FEP** | | **CTX** | | **TE** | | **AMC** | | **ESBL** |
| --- | --- | --- | --- | --- | --- | --- | --- | --- | --- | --- | --- | --- | --- | --- | --- | --- | --- | --- | --- | --- | --- | --- | --- | --- | --- |
|  |  |  |  |  |  |  |  |  |  |  |  |  |  |  |  |  |  |  |  |  |  |  |  |  |  |
| 201809183.4 | 0 | R | 35 | S | 0 | R | 23 | S | 28 | S | 27 | S | 20 | S | 18 | I | 18 | R | 0 | R | 0 | R | 19 | S | Positive |
| 201809183.4-t | 0 | R | 44 | S | 0 | R | 30 | S | 32 | S | 34 | S | 28 | S | 19 | I | 22 | I | 12 | R | 30 | S | 20 | S | Positive |
| 201809181.3 | 0 | R | 14 | R | 0 | R | 0 | R | 29 | S | 0 | R | 23 | S | 24 | S | 21 | I | 10 | R | 0 | R | 24 | S | Positive |
| 201809181.3-t | 0 | R | 44 | S | 0 | R | 30 | S | 33 | S | 32 | S | 24 | S | 21 | S | 23 | I | 13 | R | 30 | S | 23 | S | Positive |
| 2018081457.3 | 0 | R | 27 | S | 0 | R | 0 | R | 27 | S | 17 | S | 20 | S | 21 | S | 20 | I | 10 | R | 8 | R | 23 | S | Positive |
| 2018081457.3-t | 0 | R | 44 | S | 0 | R | 30 | S | 33 | S | 32 | S | 25 | S | 22 | S | 21 | I | 10 | R | 30 | S | 24 | S | Positive |
| 2018082847.3 | 0 | R | 26 | S | 0 | R | 0 | R | 33 | S | 0 | R | 20 | S | 22 | S | 21 | I | 11 | R | 0 | R | 24 | S | Positive |
| 2018082847.3-t | 0 | R | 44 | S | 0 | R | 30 | S | 33 | S | 33 | S | 24 | S | 23 | S | 22 | I | 10 | R | 30 | S | 24 | S | Positive |
| 2018081440.2 | 0 | R | 24 | S | 0 | R | 0 | R | 29 | S | 0 | R | 19 | S | 21 | S | 19 | I | 0 | R | 0 | R | 24 | S | Positive |
| 2018081440.2-t | 0 | R | 44 | S | 0 | R | 27 | S | 32 | S | 34 | S | 24 | S | 23 | S | 19 | I | 0 | R | 30 | S | 23 | S | Positive |
| 2018092531.2 | 10 | R | 36 | S | 0 | R | 22 | S | 29 | S | 19 | S | 22 | S | 14 | R | 22 | I | 12 | R | 24 | S | 22 | S | Positive |
| 2018092531.2-t | 0 | R | 44 | S | 0 | R | 28 | S | 32 | S | 20 | S | 28 | S | 19 | I | 23 | I | 11 | R | 30 | S | 22 | S | Positive |
| 201809183.3 | 0 | R | 33 | S | 0 | R | 24 | S | 29 | S | 25 | S | 21 | S | 19 | I | 18 | R | 0 | R | 0 | R | 17 | I | Positive |
| 201809183.3-t | 0 | R | 44 | S | 0 | R | 30 | S | 32 | S | 33 | S | 25 | S | 18 | I | 20 | I | 10 | R | 28 | S | 21 | S | Positive |
| 2018091176.5 | 0 | R | 12 | R | 0 | R | 25 | S | 28 | S | 17 | S | 20 | S | 17 | R | 18 | R | 10 | R | 0 | R | 18 | S | Positive |
| 2018091176.5-t | 0 | R | 44 | S | 0 | R | 30 | S | 33 | S | 34 | S | 26 | S | 16 | R | 18 | R | 10 | R | 30 | S | 21 | S | Positive |
| 2018092511.2 | 0 | R | 0 | R | 0 | R | 22 | S | 29 | S | 26 | S | 20 | S | 24 | S | 23 | I | 0 | R | 0 | R | 22 | S | Positive |
| 2018092511.2-t | 0 | R | 44 | S | 0 | R | 28 | S | 33 | S | 33 | S | 26 | S | 23 | S | 22 | I | 10 | R | 29 | S | 22 | S | Positive |
| 2018091166.4 | 0 | R | 25 | S | 0 | R | 0 | R | 29 | S | 0 | R | 20 | S | 27 | S | 25 | S | 12 | R | 0 | R | 21 | S | Positive |
| 2018091166.4-t | 0 | R | 44 | S | 0 | R | 30 | S | 33 | S | 35 | S | 24 | S | 26 | S | 26 | S | 13 | R | 30 | S | 22 | S | Positive |
| 2018091864.1 | 0 | R | 36 | S | 0 | R | 0 | R | 30 | S | 0 | R | 12 | R | 27 | S | 25 | S | 14 | R | 9 | R | 20 | S | Positive |
| 2018091864.1-t | 0 | R | 44 | S | 0 | R | 0 | R | 33 | S | 34 | S | 11 | R | 27 | S | 26 | S | 13 | R | 30 | S | 20 | S | Positive |
| 2018091135.3 | 0 | R | 0 | R | 0 | R | 0 | R | 29 | S | 0 | R | 0 | R | 24 | S | 22 | I | 9 | R | 0 | R | 17 | I | Positive |
| 2018091135.3-t | 0 | R | 44 | S | 0 | R | 30 | S | 33 | S | 34 | S | 25 | S | 23 | S | 21 | I | 10 | R | 28 | S | 22 | S | Positive |
| 2018090418.2 | 0 | R | 32 | S | 0 | R | 24 | S | 28 | S | 28 | S | 20 | S | 21 | S | 21 | I | 0 | R | 26 | S | 19 | S | Positive |
| 2018090418.2-t | 0 | R | 44 | S | 0 | R | 30 | S | 32 | S | 33 | S | 26 | S | 22 | S | 21 | I | 0 | R | 30 | S | 23 | S | Positive |
| 2018090458.2 | 0 | R | 33 | S | 0 | R | 24 | S | 29 | S | 28 | S | 22 | S | 21 | S | 20 | I | 0 | R | 26 | S | 20 | S | Positive |
| 2018090458.2-t | 0 | R | 44 | S | 0 | R | 28 | S | 32 | S | 35 | S | 24 | S | 24 | S | 22 | I | 10 | R | 30 | S | 23 | S | Positive |
| 2018081445.5 | 0 | R | 13 | R | 0 | R | 0 | R | 30 | S | 18 | S | 20 | S | 20 | I | 19 | I | 11 | R | 9 | R | 22 | S | Positive |
| 2018081445.5-t | 0 | R | 44 | S | 0 | R | 30 | S | 33 | S | 32 | S | 25 | S | 18 | I | 24 | I | 12 | R | 30 | S | 22 | S | Positive |
| 2018081441.5 | 0 | R | 0 | R | 0 | R | 0 | R | 29 | S | 0 | R | 10 | R | 23 | S | 19 | I | 10 | R | 0 | R | 16 | I | Positive |
| 2018081441.5-t | 0 | R | 44 | S | 0 | R | 30 | S | 33 | S | 35 | S | 25 | S | 22 | S | 20 | I | 11 | R | 30 | S | 22 | S | Positive |
| 2018081453.2 | 0 | R | 14 | R | 0 | R | 0 | R | 32 | S | 0 | R | 14 | I | 28 | S | 29 | S | 18 | R | 0 | R | 25 | S | Positive |
| 201810092.3 | 11 | R | 12 | R | 0 | R | 26 | S | 32 | S | 0 | R | 13 | I | 29 | S | 29 | S | 18 | R | 0 | R | 26 | S | Positive |
| 2018081445.4 | 0 | R | 13 | R | 0 | R | 0 | R | 32 | S | 0 | R | 13 | I | 25 | S | 25 | S | 15 | R | 0 | R | 23 | S | Positive |
| 2018102322.3 | 9 | R | 14 | R | 0 | R | 0 | R | 28 | S | 0 | R | 12 | R | 26 | S | 26 | S | 16 | R | 0 | R | 23 | S | Positive |

*t: transconjugant. AMC: amoxicillin-clavulanate; AM: ampicillin; CZ: cefazolin; CAZ: ceftazidime; CTX: cefotaxime; FEP: cefepime; C: chloramphenicol; CIP: ciprofloxacin; GM: gentamicin; IPM: imipenem; TE: tetracycline; SXT: trimethoprim-sulfamethoxazole. R: resistant, I: intermediate, S: susceptible.*

**Table S2.** Number of SNPs and difference length between plasmids that were sequenced in duplicate and plasmids carried by isolates of clonal relationships

|  | **Plasmid ID** | **Plasmid length (bp)** | **Difference length (bp)** | **Difference length (%)** | **SNPs (nt)** | **SNPs (%)** |
| --- | --- | --- | --- | --- | --- | --- |
| **Duplicate 1** | p201809183.3 | 71428 | 89 | 0.12 | 41 | 0.06 |
|  | p201809183.3 | 71339 |  |  |  |  |
| **Duplicate 2** | p2018091864.1 | 125759 | 193 | 0.15 | 138 | 0.11 |
|  | p2018091864.1 | 125566 |  |  |  |  |
| **Duplicate 3** | p2018081440.2 | 94894 | 16 | 0.02 | 23 | 0.02 |
|  | p2018081440.2 | 94878 |  |  |  |  |
|  |  | **MEAN** |  | **0.10** |  | **0.06** |
|  |  |  |  |  |  |  |
| **CR - 0 SNPs** | p2018090418.2 | 121366 | 234 | 0.19 | 28 | 0.02 |
|  | p2018090458.2 | 121132 |  |  |  |  |
| **CR- 90 SNPs** | p2018091135.3 | 127219 | 21295 | 16.74 | 209 | 0.20 |
|  | p2018081441.5 | 105924 |  |  |  |  |

*CR: clonal relationship (>100 SNPs on core genome).*
